# Comparison of Optical Coherence Tomography Angiography to Indocyanine Green Angiography and Slit Lamp Photography for Corneal Vascularization in an Animal Model

**DOI:** 10.1038/s41598-018-29752-5

**Published:** 2018-07-31

**Authors:** Tisha P. Stanzel, Kavya Devarajan, Nyein C. Lwin, Gary H. Yam, Leopold Schmetterer, Jodhbir S. Mehta, Marcus Ang

**Affiliations:** 1 Singapore Eye Research Institute, 169856 Singapore, Singapore; 2 Eye-ACP, Duke-NUS Graduate Medical School, 169857 Singapore, Singapore; 3 Nanyang Technological University, 639798 Singapore, Singapore; 4 Department of Clinical Pharmacology, Medical University of Vienna, 1090 Vienna, Austria; 5 Center for Medical Physics and Biomedical Engineering, Medical University of Vienna, 1090 Vienna, Austria; 6 Singapore National Eye Center, 168751 Singapore, Singapore

## Abstract

Corneal neovascularization (CoNV) could be treated by novel anti-angiogenic therapies, though reliable and objective imaging tools to evaluate corneal vasculature and treatment efficacy is still lacking. Optical coherence tomography angiography (OCTA) –currently designed as a retinal vascular imaging system— has been recently adapted for anterior-segment and showed good potential for successful imaging of CoNV. However, further development requires an animal model where parameters can be studied more carefully with histological comparison. Our study evaluated the OCTA in suture-induced CoNV in a rabbit model compared to indocyanine green angiography (ICGA) and slit-lamp photography (SLP). Overall vessel density measurements from OCTA showed good correlation with ICGA (0.957) and SLP (0.992). Vessels density by OCTA was higher than ICGA and SLP (mean = 20.77 ± 9.8%, 15.71 ± 6.28% and 17.55 ± 8.36%, respectively, *P* < 0.05). OCTA was able to depict CoNV similarly to SLP and ICGA, though it could better detect small vessels. Moreover, the depth and growth of vessels could be assessed using en-face and serial-scans. This study validated the OCTA in a rabbit model as a useful imaging tool for translational studies on CoNV. This may contribute to further studies on OCTA for anterior-segment including serial evaluation of emerging anti-angiogenic therapies.

## Introduction

Corneal neovascularization (CoNV) is a pathological condition which compromises the immune privilege state of the cornea, reduces corneal transparency, causes vision loss and subsequently increases the rate of graft rejection after corneal transplantation^1,2^. Treatment of CoNV includes topical steroids to inhibit inflammatory cell chemotaxis and cytokines, induced vessels stenosis by electrocoagulation or laser, and in resistant cases, surgical removal might be indicated^3^. Recent knowledge of cellular and molecular pathogenesis of CoNV leads to flourishing of novel target anti-angiogenic approaches—using anti-VEGF (vascular endothelial growth factor) antibody, VEGF trap or silencing RNA^4–6^. In order to evaluate these new treatment outcomes - such as quantitative measurement of vessel inhibition or regression, a reliable and effective imaging system is in need^7^.

Currently, slit-lamp photography (SLP) or videography with image analysis software is commonly used to quantify the area of CoNV in both clinical and experimental settings^8,9^. The analysis is time-consuming and color photographs have limited visualization of vessels in the presence of corneal edema, deposits or scar – even more so for very small caliber or deeper layer vessels^10^. Moreover, the accuracy and repeatability of slit-lamp photography for CoNV are still under validation^11,12^. Fluorescein and indocyanine green angiography techniques have demonstrated better vessel delineation than slit-lamp photograph despite the presences of corneal scars^13^. The videographic function showing blood flow and leakage is a useful tool to evaluate dynamic vessel properties^13^. However, both techniques are invasive and expose subjects to common gastrointestinal side effect or serious adverse reaction like anaphylactic shock^14–16^. Another limitation of the above procedures is that they measure the vascularized area only in two dimensions. Confocal imaging may provide higher resolution for corneal imaging, but requires direct contact with the cornea and an extremely small field of view which may have limited use in extensive areas of CoNV. As such, visualization of corneal neovascularization over the entire cornea would require stitching of many individual images, which is a time-consuming procedure. Confocal microscopy and two-photon microscopy could capture three-dimensional microscopic images of cornea vessels in *in-vivo* setting – but are only evaluated primarily in small animals thus far^17,18^.

Optical coherence tomography (OCT) angiography is an additional algorithm of the widely used OCT method^19^, which assesses posterior and anterior segment vessels in a rapid, non-invasive, non-contact manner^20–22^. OCT angiography (OCTA) has been shown to surpass the limitation and disadvantages of injected-dye angiographies and has been shown to demonstrate more detail of superficial vessels and view deeper layers of retinal vascular network than a conventional angiography^23^. We have previously described the use of OCTA for corneal vascularization in patients, demonstrating good repeatability and reproducibility^24,25^. This technique showed good agreement with indocyanine green angiography (ICGA) for vessel delineation and could quantitatively monitor vascularized area after antiangiogenic treatment^26–28^. Furthermore, it has been suggested that OCTA could be useful to study anterior segment vascular conditions like ocular inflammatory disorders, infectious keratitis, limbal stem cells deficiency including ocular surface neoplasia or corneal graft rejection^27^.

Despite previous clinical assessment of OCTA in humans, its application in animal model is useful for further understanding disease mechanisms, knowledge in molecular pathogenesis and assessment of novel therapeutic approaches in translational work. The rabbit cornea model was chosen given its similarity to the structure of the human cornea, while suture-induced cornea neovascularization provides a more extensive vascular network for the imaging study. Moreover, motion artifacts are significantly minimized in an animal model study. This proof of concept study was conducted following our previous work in humans, and in an effort to establish OCTA as an imaging tool for animal models of CoNV for future translational studies.

To our knowledge, there has been no prior evaluation of OCTA for CoNV in an animal model. Hence, we assessed first time, the use of OCTA for depicting and measuring corneal vascularization in a rabbit model, as well as comparisons to ICGA and slit-lamp photography.

## Results

**Figure 1:**
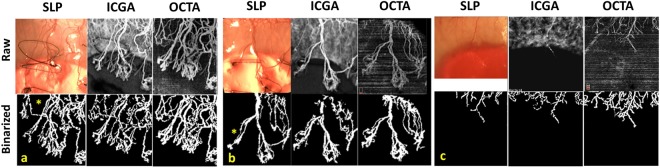
Examples of matched raw and processed images obtained from slit lamp photography (SLP), indocyanine green angiography (ICGA) and optical coherence tomography angiography (OCTA). Raw images (top row) from SLP (left column), ICGA (middle column) and OCTA (right column) depict vessels from the same region of interest (ROI), approximately 3 × 3 mm^2^, according to OCTA capture scale. Note the less prominent background iris vessels present in OCTA compared to SLP and ICGA images. Processed or binarized images (bottom row) show that OCTA can detect and delineate vessels like other two techniques. The signals from small caliber vessels (the area is marked with * in the image set a and b) were reduced during image analysis and could not be detected in the processed SLP images. The same vessels are more distinct in OCTA than ICGA. Image set c shows regressed vessels which are barely detected by raw images (top row) of SLP and ICGA but clearly present in OCTA. Binary images (bottom row) show clearly the superior detection of regressed vessels by OCTA than ICGA and SLP.

**Figure 2:**
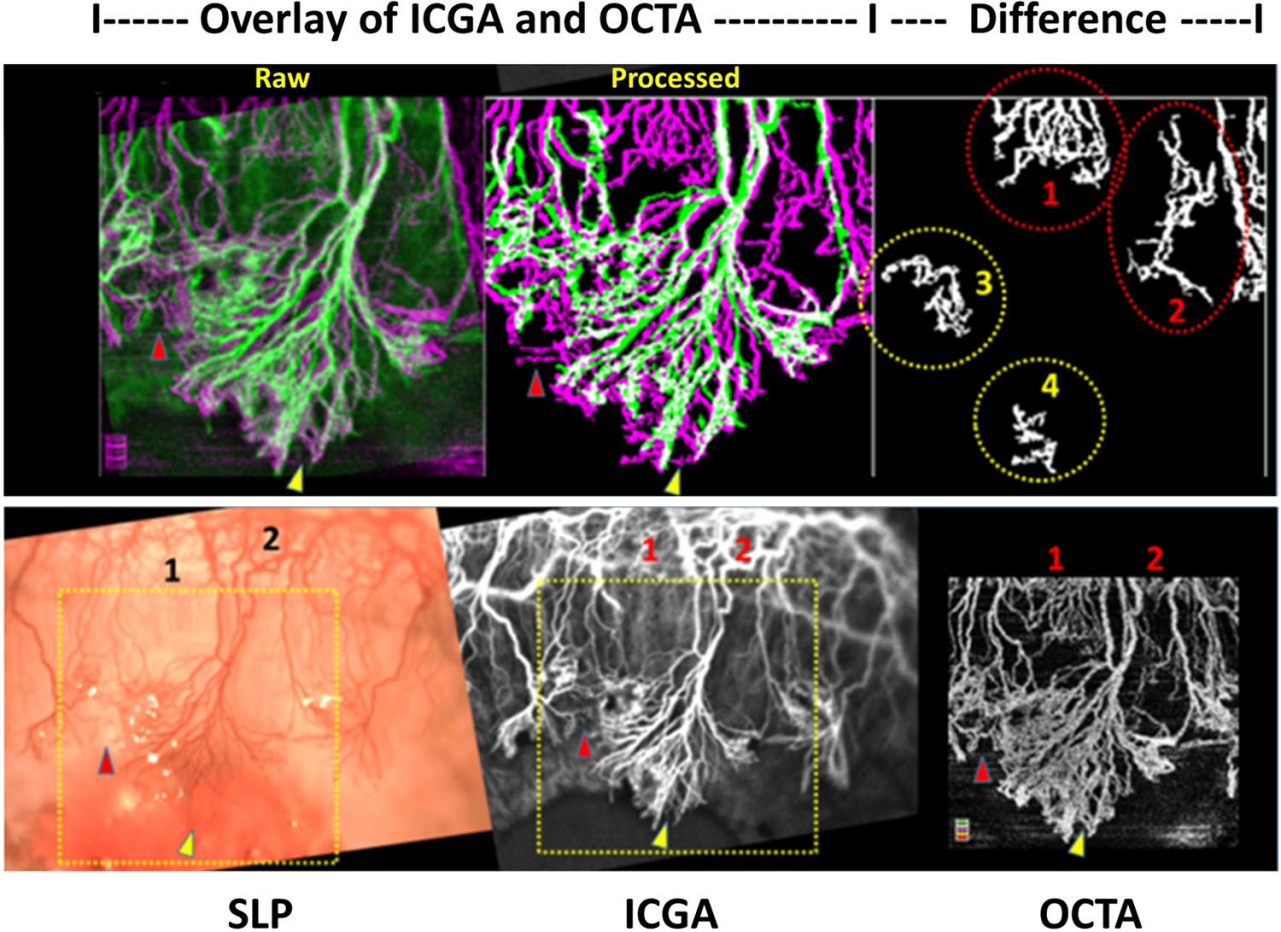
Overlay matched images show the difference of vessels delineation between indocyanine green angiography (ICGA) and optical coherence tomography angiography (OCTA). Raw and processed (binarized) images from ICGA (green color) and OCTA (pink color) of rabbit 5 at week 6 are overlaid for comparison (approximate image scale 3 × 3 mm^2^). The difference between the two images is presented as binary images (top right) which show the extra vessels detected by OCTA. Additional vessels in area 1 and 2 (red circles) are small caliber vessels which are barely detected in the corresponding color slit-lamp photograph (SLP) and ICGA (bottom row). Vessels in area 3 and 4 (yellow circles) can be detected by all image techniques (red and yellow Δ) but threshold out as background in processed images of SLP and ICGA. Segmentation allows for removal of artifacts and reduction of image interference from the iris vessels in OCTA than in SLP and ICGA (bottom row).

**Figure 3:**
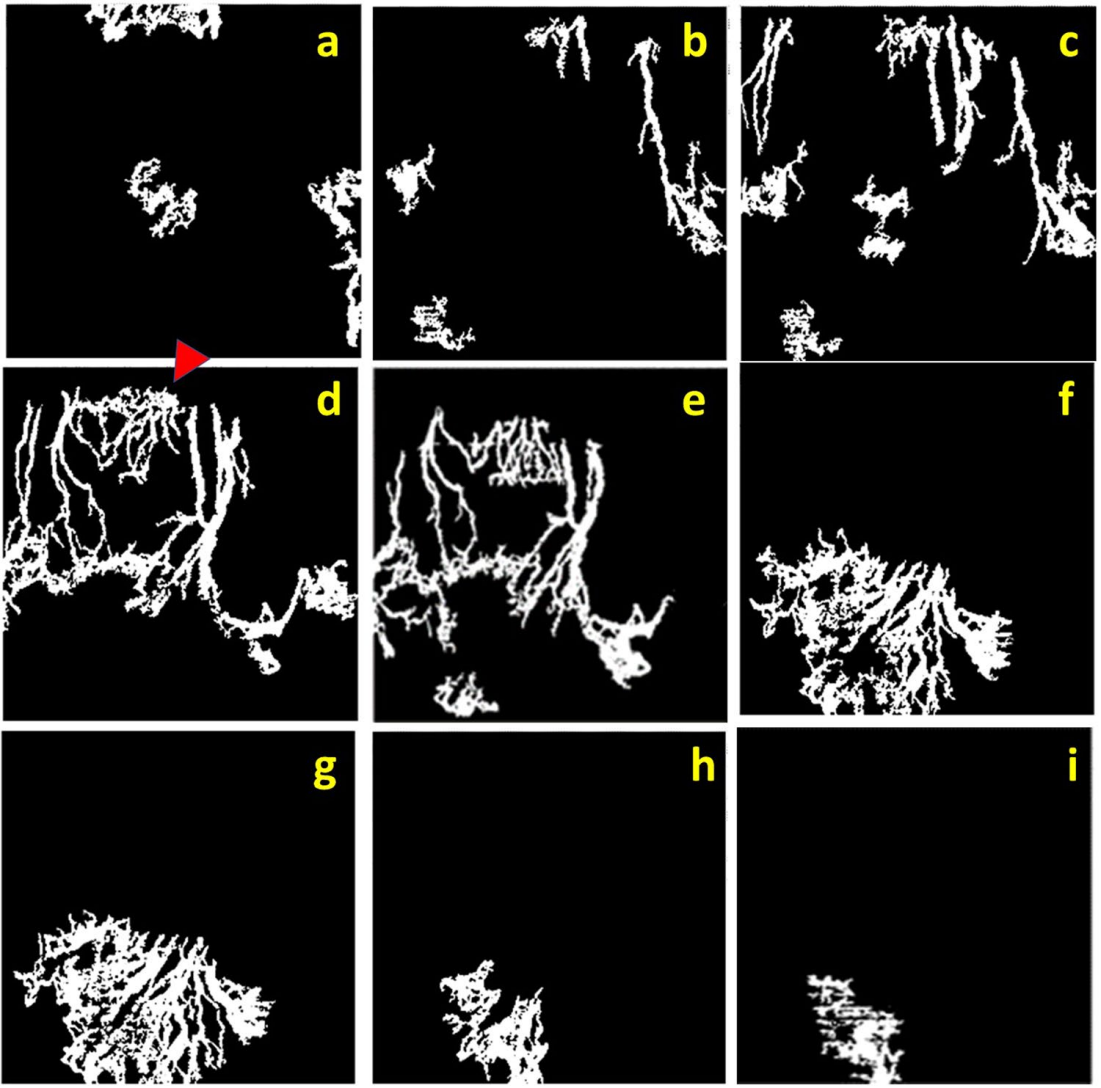
Sequence of processed ‘en face’ optical coherence tomography angiography (OCTA) demonstrates vessels at different corneal depths. A series of coronal ‘en face’ corneal sections from subepithelial (**a**) to mid stroma layer (**i**) of rabbit 5 at week 6, every 20 microns interval, depicting corneal vessels of the same rabbit 5 at week 6 as in Fig. 2. The red Δ indicates the same vessels in area 1 of Fig. 2, which are present in superficial layers (**d**,**e**) whereas the fan-like vessels are present in the deeper layers (**f**,**g**,**h**). Note, discontinuity of the vascular network and overlapping of same vessels in different frames caused by unparallel segmentation, which stems from the natural three dimension growth of vessels and steep rabbit corneal curvature.

**Figure 4:**
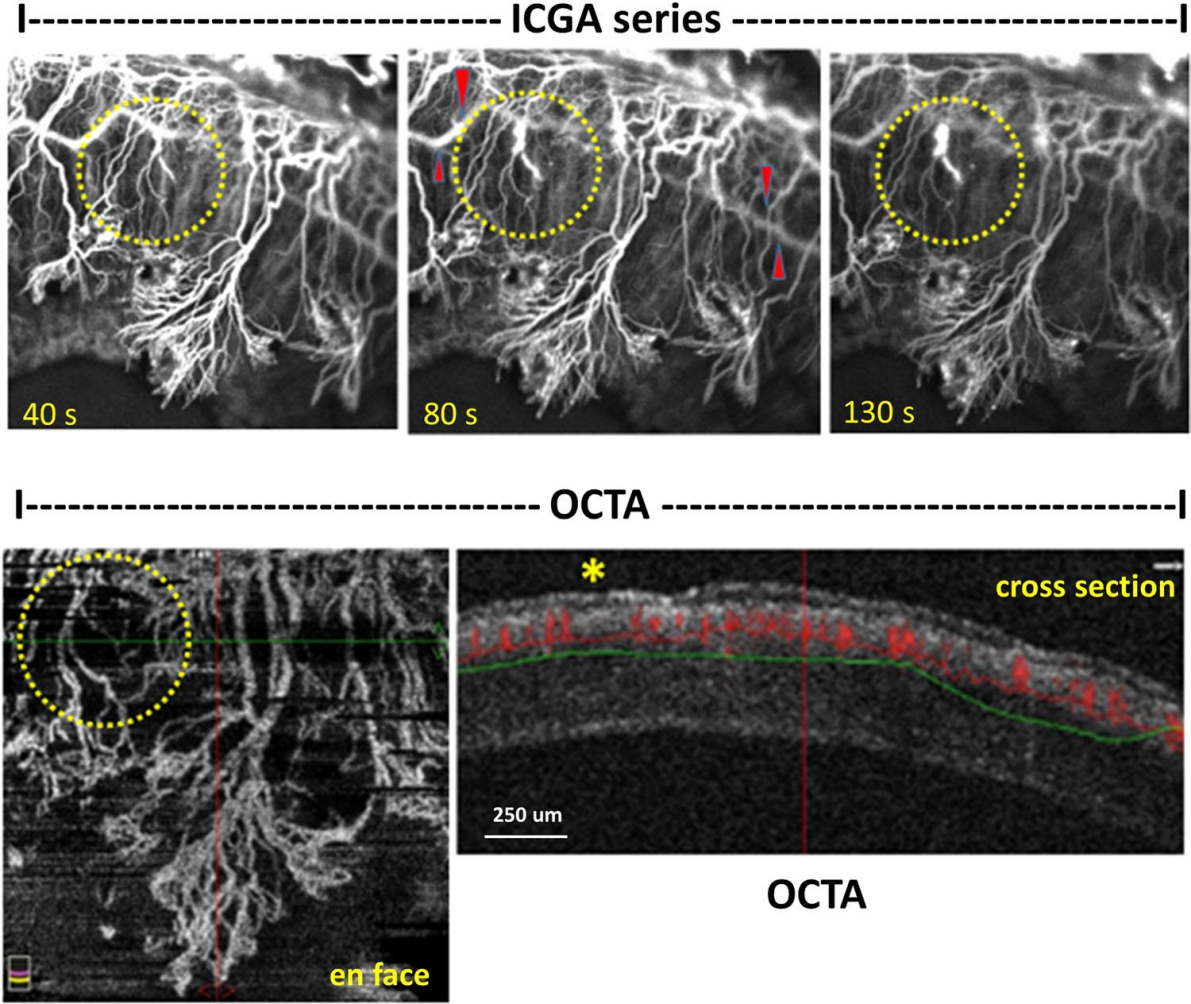
Stained vessels in indocyanine green angiography (ICGA) image series correspond with ‘no signal’ vessels in optical coherence tomography angiography (OCTA). A series of ICGA images (top row, 40, 80 and 130 seconds, respectively) show leakage from corneal vessels (in yellow circles), increasing in intensity over time, while the overall vessels signal intensity reduces. Note the strong background signal of horizontal iris vessels (indicated by red Δ) interferes the ICGA corneal vessels. ‘En face’ OCTA scan (bottom left) detects the same vessels in ICGA. Corresponding cross-sectional B scan (bottom right) shows ‘no flow’ of blood (marked by *) in the leakage vessels. The cross-sectional plane is indicated by a green horizontal cross line in the en-face scan. Note, cross-sectional scan shows that all induced vessels, represented by a red signal of the blood flow, are located above the green horizontal line, which indicates the approximate 200 microns depth of scan, thus in the anterior corneal stroma.

A total of 240 OCTA scans (5 scans/rabbit/week) were taken during the 8 weeks study period, and images with CoNV were analyzed from week 3 onwards where the peak of CoNV developed beyond 3 millimeters from the limbus into the cornea. Sets of matched regions of interest (ROI) were compared amongst all three imaging techniques (OCTA, ICGA, and SLP) – Fig. 1. We noted that the OCTA could capture small caliber vessels, and regressed vessels, which were not detected by slit-lamp and better delineated as compared to ICGA – Fig. 2. We also found that the OCTA images could determine the depth of the corneal vessels, which cannot be done with SLP and ICGA – Fig. 3. Moreover, there was no interference of vessel ICG leakage or background iris vessel imaging as seen in ICGA images compared to OCTA – Fig. 4.

### Vessels density measurements

OCTA, ICGA and SLP images were used to compare the vessel density measurements across the three techniques. Overall vessel density measurements from OCTA showed good correlation values with ICGA (0.957) and SLP (0.992). The mean vessel density by OCTA, 20.77 ± 9.8%, was significantly greater than the values by ICGA and SLP, 15.71 ± 6.28% and 17.55 ± 8.36%, respectively, *P* < 0.05. There was no difference in the measurements of vessel density between ICGA and SLP, *P* > 0.05.

**Figure 5:**
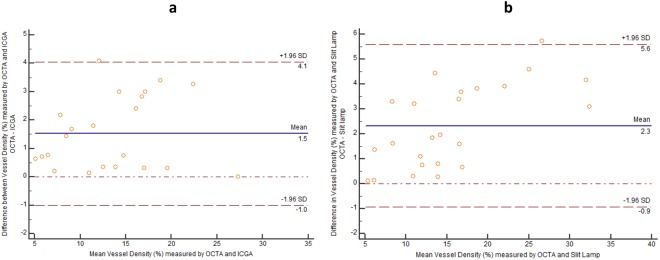
Vessels density measurements from optical coherence tomography angiography (OCTA) compared with indocyanine green angiography (ICGA) and slit lamp photography (SLP). The Bland-Altman plot between the differences of vessels density measurements from OCTA and ICGA (y-axis) in (**a**) (OCTA and SLP in **b**) against the average vessels density measurements of the 2 methods (x-axis) —showing good agreement of vessels density obtained from both imaging methods. The measurements of the density of corneal vessels within the region of interest were obtained from 22 sets of matched OCTA and ICGA images and 23 sets of matched OCTA and SLP images. The mean difference between OCTA and ICGA = 1.5 ± 2.6% (95% CI, 0.9526 to 2.0957%, *P* < 0.0001). Limits of agreement (±1.96 SD) = 4.1 and −1.0%. The mean difference between OCTA and SLP = 2.3 ± 3.3% (95% CI, 1.5135 to 2.8416%, *P* < 0.0001). Limits of agreement (±1.96 SD) = 5.6 and −0.9%. Solid line = mean of the difference. Short dashed line = reference zero. Long dashed line = upper and lower 95% limits of agreement (mean + 1.96 SD, mean −1.96 SD). SD = standard deviation of the mean difference.

We found good agreements of vessels density measurements between OCTA versus ICGA and OCTA versus SLP, analyzed using Bland-Altman plots. The vessels density measurement by OCTA was higher than the values by ICGA (1.5%, 95% CI, 0.9526 to 2.0957%, *P* < 0.0001) and the values of SLP (2.2%, 95% CI, 1.5135 to 2.8416%, *P* < 0.0001) – Fig. 5. Good agreement of vessel density measurements between ICGA and SLP was also observed with a mean difference of 0.02 ± 0.07% (95%CI: −0.0413 to 0.0053%), *P* = 0.11 (10 sets of Supplementary Figure images, data in supplement).

### Vessels growth density by serial OCTA

**Figure 6:**
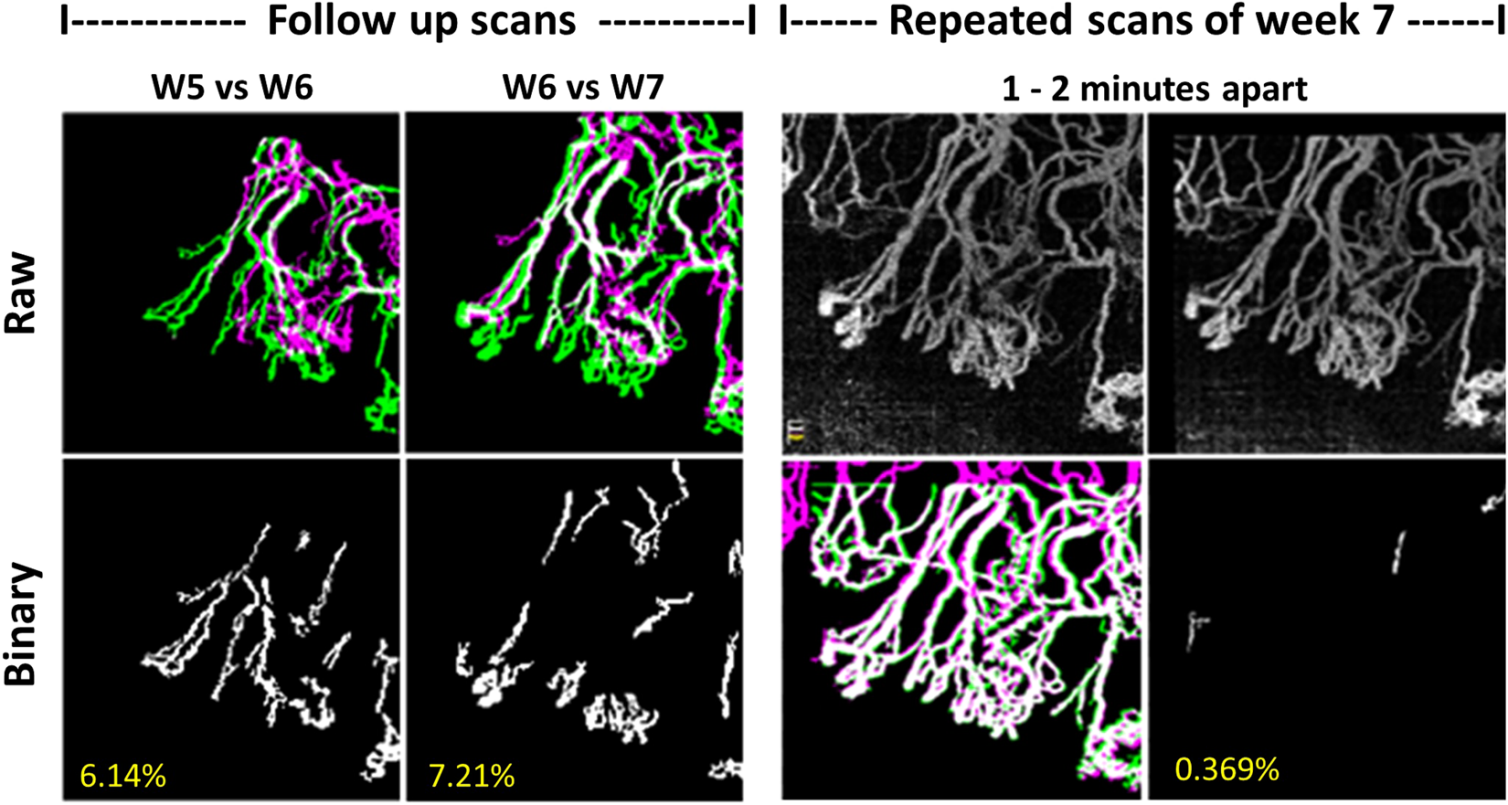
Example to illustrate vessel growth density measurements using serial optical coherence tomography angiography (OCTA) imaging. An example of vessel growth analysis uses the overlay of binarized images of the new vessels (green) in the recent follow-up over the existing vessels (pink) from the previous follow-up. The binarized images show new vessels growth (vessels density difference) of week 6 from 5 (left, growth density 6.14%) and week 7 from 6 (right, growth density 7.21%) in the same region of interest (ROI). In order to illustrate that these detected changes are significant, we repeated OCTA scans in the same ROI and performed binary overlay analysis of the repeated scans that were taken at the same time point (1–2 minutes apart). Vessel growth density difference - computed between binarized repeated scans with vessel density of 0.369% (bottom right).

Serial OCTA was able to successfully measure the increase in vessel density during the follow-up period in our study. The change in vessel growth density from baseline to final follow-up OCTA images were significantly greater than the mean standard error from repeated vessel density measurements in the same ROI of the same eye, 4.184 ± 2.063% vs. 0.4592 ± 0.065%, *P* < 0.001. – Fig. 6.

### Cornea whole mount immunohistochemistry

**Figure 7:**
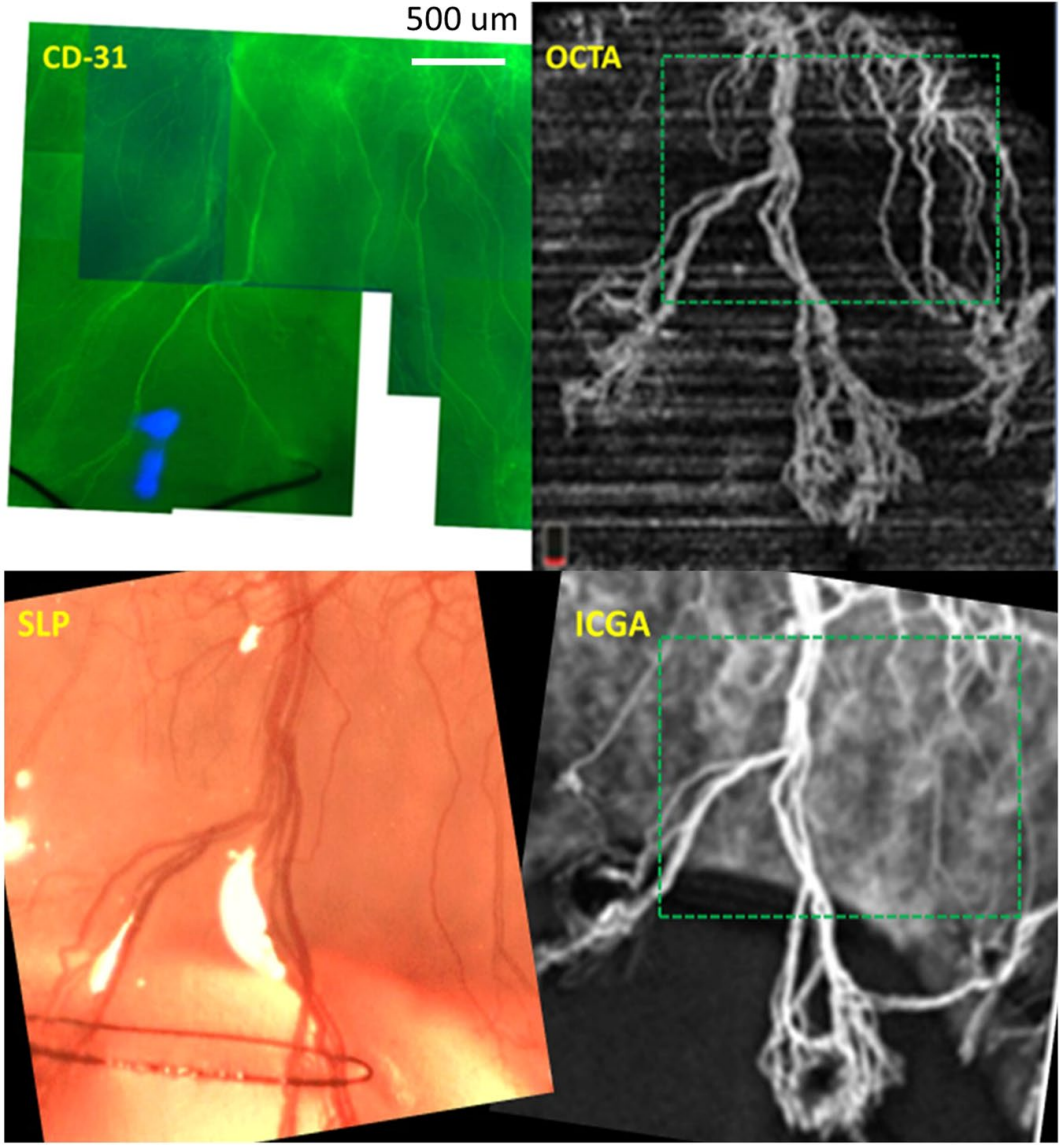
Example of whole mount cornea showing CD-31 stained blood vessels in suture-induced cornea neovascularization (CoNV) model captured by fluorescence biomicroscopy. A portion of whole mount cornea of rabbit 3 (same as Fig. 1 set 2) shows blood vessels, stained by CD-31. The corresponding area imaged with optical coherence tomography angiography (OCTA), slit-lamp photography (SLP) and indocyanine green angiography (ICGA) images are matched. (Bordered by green dashed squares in OCTA and ICGA). Note, different angle of image acquisition alters position and length of the vessels in each image.

Blood vessels in wholemount cornea tissues (3 pieces/rabbit) were successfully stained with CD-31 and viewed by fluorescence microscopy. Strong background staining due to corneal thickness was noticed in all sections. CD-31 stained blood vessels corresponded with images from SLP, ICGA, and OCTA. Approximately 10–15 immuno-stained images (scale 200 μm) were required to be manually stitched together in able to match one part of 3 × 3 mm image scan. Immuno-stained images could capture the higher detail of vessels including vessel wall and small caliber vessels. The depth of image capture was limited, and only vessels in a narrow depth of field were in focus. Due to high magnification, the field of view was limited to a small part of full 3 × 3 mm scan. – Fig. 7.

## Discussion

Since the recent advancement of the technology and its availability for clinical implementation, the interest of OCTA adaptation for the anterior segment has grown rapidly^29^. Most of the published work in humans thus far, have suggested that OCTA may be useful in the clinical setting. However, evaluation of novel therapeutic approaches for corneal neovascularization needs to be done in animal models. Our previous clinical studies have reported the use of the built-in software within the OCTA system, originally designed for retinal blood flow analysis, to assess the corneal vessels^24^. This subsequent animal study using the same split-spectrum amplitude-decorrelation angiography (SSADA), has confirmed that the OCTA is also able to delineate corneal vascularization in a corneal animal model^26^.

This study is, to our knowledge, the first report of using OCTA to evaluate cornea neovascularization (CoNV) and compared with indocyanine green angiography (ICGA) and slit-lamp photography (SLP) in an animal model. Our results suggest that the anterior segment adapted OCTA system may delineate corneal vessels better than ICGA and SLP in the animal model, especially vessels with small caliber, located in deeper layers and particularly detecting regression after treatment (Figs 1 and 2). Moreover OCTA provides good agreement of vessels density measurements with the other two techniques; however, it may overestimate vessel density than the ICGA and slit-lamp images, which needs to be taken into account if used in future studies (1.5 and 2.3% of total image capture area respectively).

The measurement disparity between OCTA and ICGA in this recent report was greater than what we observed in the previous clinical study (0.135 mm^2^ vs. 0.03 mm^2^). The comparison was done by converting the vessel density value from the percentage of total image capture area to square millimeters by multiplying the value with the scan area acquisition. This disparity might be caused by the better image acquisition in an animal model—less motion artifacts, relatively large area of CoNV, higher number (N) of studied images and less limited handling time. The disparity is even more when we compared OCTA with slit-lamp photography. This finding was consistent with a previous report, which compared dye angiographies with slit-lamp for CoNV quantification^13^. The higher vessels density measurement by OCTA could be due to three possible explanations: Firstly, the better optical transverse resolution of OCTA could detect smaller vessels, which are missed by other two techniques^30,31^. Secondly, OCTA has a higher depth of focus which allows for detection of vessels from deeper layers than other devices. Thirdly, the signal of small vessels in ICGA and slit lamp tends to be removed together with relatively high iris background during image binarization.

The ability of OCTA to detect small vessels that are not visible by ICGA and slit lamp depends on the resolution of the OCT system. The axial resolution of the OCTA is determined by the coherence length of the light source and in the order of 5 μm. The lateral resolution depends on the spot size of the laser beam on the cornea and the oversampling ratio^32,33^. Hence, the smaller vessel in the order of 10 μm may be visible in smaller scanning patterns of 3 × 3 mm, whereas larger scanning patterns of 12 × 12 mm may only allow for visualization of the vessel with a diameter of 20 μm and above. Obviously, neither slit lamp nor ICGA provides depth resolution. With the latter technique, the dye is used for contrasting blood vessels, but lateral resolution may be reduced due to leakage.

We studied two new parameters, vessel density percentage and vessel growth density that provide quantitative tools to assess and follow up on corneal angiogenesis from different imaging techniques. Using these parameters (Fig. 6), we found that the OCTA is able to detect a weekly vessel growth. Moreover, this study confirmed the usefulness of the coronal ‘en face’ function to locate the depth of corneal vessels as previously described^25,26^ (Fig. 3). With cross-sectional angiography modality—showing the signal of blood flow in corneal vessels—the depth and flow of vessels could be assessed (Fig. 4); and we correlated the area of CoNV imaging with histological confirmation of vascularization using CD-31, a non-specific vessel endothelial marker.

In addition to the advantage of OCTA being able to perform a rapid, non-contact volumetric scan of the cornea^20,29^, this study has emphasized the usefulness of OCTA for detection, quantification, as well as sequential follow up of corneal vascularization, particularly small caliber vessels or vessels regression during treatment^34^. This successful proof of concept study could support the future use of OCTA for the anterior segment in an animal model, which will further improve our understanding of pathologies and treatment related to corneal vascularization. OCTA could also greatly beneficial for the study of ocular inflammatory diseases^35^, corneal graft vascularisation, anterior segment tumor vascularity, secondary or neovascular glaucoma or limbal stem cell deficiency^36,37^.

Despite the advantages and technical improvements in OCTA technology, it is important to note the limitations of this imaging technique. Originally, the OCTA was designed for imaging of retinal vasculature; it is optimized for a relatively flat surface when compared to the curvature of corneal tissue. An improper eye positioning or a large scanning area will create a non-perpendicular image capture thereby causing projection artifacts, as well as non-parallel segmentation. As a result, superficial vessels may appear thicker^38^ and vessels caliber discontinuity occurs in coronal en-face sections. During image capture, a subtle movement such as animal breathing can cause motion artifacts. The artifacts or improper software correction may lead to vessels duplication, residual motion line, and also vessels discontinuity^29^. We observed that it requires a learning curve to capture desirable images—which are perpendicular to the corneal surface and with minimal motion artifacts. Ideally, the induced vessels should be in any quadrant of the cornea but with the restriction of imaging position in the rabbit, similar to another study, we placed the sutures, then captured the images only in the superior cornea^39^. In the current phase of development for anterior segment imaging, the segmentation still requires manual correction in a minority of cases. The workload can, however, be strongly reduced with techniques such as image registration, artifact reduction and morphological processing. This software approach can compensate for some movement artifacts, but can introduce artifacts of its own, including loss of detail in the image despite a high signal score. This might be due to trade-off in the application of smoothing filters such as Gaussian filter in the processing and due to different lateral spot sizes of the imaging system. Furthermore, the lower resolution of the OCTA images may also lead to the thicker vessel density in comparison to ICGA and slit-lamp images. Fundamentally horizontal image of OCTA scan can easily miss vertical growth vessels. Importantly, manual background removal during image processing could contribute to inter- or intrapersonal variation, bias or error. Lastly, though its distinct benefit over ICGA and slit-lamp, OCTA has its own limitations^40^. Unlike dye angiography, OCTA has no leakage, which is important to assess vessels maturity and vascular occlusion. OCTA also does not show blood flow and cannot truly identify the feeder vessels, which is essential for diathermy treatment^41^.

In conclusion, our pilot study suggests that the OCTA is a useful imaging technique for corneal vascularization in an animal model, and may have a potentially greater sensitivity in detecting microvasculature with a larger depth of focus. The OCTA imaging technique described is a non-invasive, fast and non-contact procedure that can evaluate corneal vessels, which is useful for future animal model-based studies for quantification and serial measurements in corneal vascularization.

## Methods

We conducted a prospective observational study using an established model of corneal vascularization^39^. The study was approved by the Institutional Animal Care and Use Committee of SingHealth. All experimental procedures were carried out in accordance with the guideline of the Association for Research in Vision and Ophthalmology for the use of animals in ophthalmic and vision research.

### Induction of corneal vascularization

A total of six male New Zealand White rabbits weighing 2.5–3.5 kg (InVivos Pte Ltd, Singapore) were used in this study. One eye per rabbit received corneal suturing under general anesthesia [intramuscular xylazine HCl (5 mg/kg) and ketamine HCl (50 mg/kg)], supplemented by topical anesthesia (0.4% oxybuprocaine HCl). A modification from previously described suture technique was performed to induce corneal vascularization^39^. Eight interrupted 10-0 non-absorbable nylon sutures (B. Braun Surgical SA, Spain) were placed at mid-stromal depth in the superior part of the cornea in an inverted triangle fashion. The outer row consisted of 3 stitches, 1 mm away from the limbus. In parallel to the outer row, the middle row consisted of 3 stitches. The inner row consisted of 2 stitches, placed in between the stitches of the middle row. All stitches were 2.5–3.5 mm in length and 1.0–1.5 mm apart from each other. Antibiotic eyedrops (tobramycin ophthalmic ointment 0.3%, Alcon Labs Inc, Texas, USA) was applied twice daily throughout the follow-up period. All 6 rabbits developed corneal vascularization in the superior quadrant of the cornea after the suture-induced experiment. The new vessels started growing from the first week and reached the peak around 3^rd^ to 4^th^ week. The stitches were stepwise removed, outer row at week 3, middle row at week 4. The innermost stitches were kept until the final follow-up. Extra stitches were placed inside the innermost row to obtain the centralized growth of vessels.

### Imaging and angiography techniques

After suture placement, all rabbits were evaluated under anesthesia on a weekly basis until week 8 and then sacrificed. The area of CoNV was documented using firstly SLP, then OCTA, and ICGA in each follow-up. Color SLP images were captured using the digital slit-lamp camera (Righton NS-2D, Tohoku Right Mfg., Miyagi, Japan) with a standard diffuse illumination (x12 to x36 magnification).

OCTA of the cornea was acquired using a split-spectrum amplitude-decorrelation angiography system (AngioVue, Optovue Inc, Fremont, California, USA) with the long corneal adaptor module (CAM-L). The scan was taken 2 times in the same area (each requiring average 4–6 seconds), ensuring good signal strength. Essentially, all eyes had 3 × 3 mm^2^ and 6 × 6 mm^2^ scans of the identified area of CoNV. The autofocus function was deactivated, and the lens were moved very close (2–3 cm) to the corneal surface before fine-tuning and manual adjustments of the focal lengths to achieve adequate focus of the CoNV. The scans had a transverse resolution of 15 μm and an axial resolution of 5 μm using a light source centered on 840 nm with a beam width of 22 μm. Coronal or ‘en face’ OCTA scan images were reconstructed from 304 × 304 A-scans captured at 70,000 scans per second^42^.

ICGA of anterior segment was performed using scanning laser ophthalmoscope (Anterior segment objective lens, HRA2, SPECTRALIS® scanning laser angiography, Heidelberg Engineering, Heidelberg, Germany). Immediately after intravenous injection of ICG dye (2.5 mg/ml vial, 2 mg/kg), multiple single-frame images of CoNV were repeatedly captured every 3 to 5 seconds using a 20° and 30° of scan angle in “Hi-Res Mode” with 100% laser power for 3 minutes. To facilitate subsequence image analysis, the image captured area was set to 5 regions–at supero-temporal, supero-central and supero-nasal for high magnification (to match with the 3 × 3 mm^2^ OCTA) and supero-temporal and supero-nasal for low magnification (to match with the 6 × 6 mm^2^ OCTA). The inferior quadrant of the corneas was not captured, as there was no induced vessel. Total of 30 OCTA scans in each week (5 scans per rabbit per week) were then matched with the serial ICGA and SLP images.

### Whole mount cornea immunohistochemistry

The excised corneas were prepared for whole mount staining using a modification from the previous reports^43–46^. In brief, after being rinsed in phosphate-buffered saline (PBS), the superior corneas were cut into 3 wedges and fixed in 4% paraformaldehyde at 4 °C overnight. Then the tissues were washed by 1% Triton X 100 in PBS and blocked with 1% Triton X 100 in 5% normal donkey serum in PBS for 1 hour. The corneas were stained overnight at 4 °C with mouse anti-rabbit monoclonal CD-31 (1:200, Abcam Singapore Pte Ltd, Singapore). The CD-31 was then detected with goat anti-mouse secondary antibody, either Alexa flour 488 (Thermo Fisher Scientific, Singapore) or Rhodamine-X (Jackson ImmunoResearch, USA). After the final washed, the cornea tissues were mounted endothelial size down and viewed by a fluorescence biomicroscopy.

### Images processing and vessel density measurements

The best SLP, ICGA and OCTA images were independently selected from 5 captured regions by same two observers (TPS and KD). The selected images were then exported from the system as Portable Network Graphics files (OCTA) and Joint Photographic Experts Group files (ICGA, SLP) for image analysis.

The captured images were converted from RGB to grayscale. Image registration was done between the images captured with ICGA, Slit Lamp, and OCTA to compensate for the difference in magnification and the angle of acquisition caused during the imaging using the intensity-based automatic image registration in MATLAB. The overlay image after registering defined the measurement region in which the vessels were processed.

In order to eliminate the speckle noise and horizontal motion artifacts, median filtering and 2-D Gaussian smoothing kernel with standard deviation one were applied to the OCTA images^47^. Top-hat filtering was performed to reduce noise and enhance linear contrast in all three image datasets. For each of the dataset, the opening of reconstruction operation, applied to the structuring element parameter, was specific to background illumination of the image. This pre-processing step sharpened the blood vessels and improve signal-to-noise ratio while preserving the image features^48^.

The aforementioned image pre-processing steps, based on an earlier study^49^, was modified for the binarization step with automated segmentation algorithm for better accuracy and performance. The image binarization was performed using an automated computed threshold and the local phase based filter instead of setting a global threshold^48,50^. The threshold was applied so the details in the part of the image with lower signal-to-noise ratio would be maintained. In the binarized processed images, white pixels represented the blood vessels and black pixels represented the background. The iris vessels background was manually removed, if needed, in the ICGA and slit lamp images. Vessel density was computed by the following equation. (Fig. 1).

$${\rm{V}}{\rm{e}}{\rm{s}}{\rm{s}}{\rm{e}}{\rm{l}}\,{\rm{d}}{\rm{e}}{\rm{n}}{\rm{s}}{\rm{i}}{\rm{t}}{\rm{y}}\,{\rm{p}}{\rm{e}}{\rm{r}}{\rm{c}}{\rm{e}}{\rm{n}}{\rm{t}}{\rm{a}}{\rm{g}}{\rm{e}}=(\int {\rm{V}}.{\rm{d}}{\rm{A}}.\,/\,\int {\rm{d}}{\rm{A}}.\,)\ast 100$$

where V = 1 for white pixels (blood vessels); V = 0 for black pixels (background) after binarization of the matched image. A is the region of interest (ROI)^51,52^.

Vessel Growth Density was computed to determine the growth percentage of vessels imaged by OCTA between every pair of consecutive follow-up scans. Successive weekly follow-up OCTA images of each rabbit were overlaid using image registration to match for ROI based on intensity. After artifact removal using similar image processing algorithm, the binarized images matched for the same ROI were derived for each rabbit. The binarized image from the previous follow-up scan was subtracted from the new consecutive follow-up binarized image. Minute vessel density changes in the subtracted binary image due to small misalignments in the images, were eliminated by applying threshold in the image area of above 2 pixel units. The vessel density percentage computed in the binary subtracted image between consecutive follow-up was defined as the vessel growth percentage at that time-point, shown in the following equation.

$$\begin{array}{c}{\rm{V}}{\rm{e}}{\rm{s}}{\rm{s}}{\rm{e}}{\rm{l}}{\rm{s}}\,{\rm{g}}{\rm{r}}{\rm{o}}{\rm{w}}{\rm{t}}{\rm{h}}\,{\rm{d}}{\rm{e}}{\rm{n}}{\rm{s}}{\rm{i}}{\rm{t}}{\rm{y}}\,{\rm{p}}{\rm{e}}{\rm{r}}{\rm{c}}{\rm{e}}{\rm{n}}{\rm{t}}{\rm{a}}{\rm{g}}{\rm{e}}\,{\rm{a}}{\rm{t}}\,{\rm{w}}{\rm{e}}{\rm{e}}{\rm{k}}\,({\rm{n}}+1)={\rm{V}}{\rm{e}}{\rm{s}}{\rm{s}}{\rm{e}}{\rm{l}}{\rm{s}}\,{\rm{d}}{\rm{e}}{\rm{n}}{\rm{s}}{\rm{i}}{\rm{t}}{\rm{y}}\,\\ {\rm{p}}{\rm{e}}{\rm{r}}{\rm{c}}{\rm{e}}{\rm{n}}{\rm{t}}{\rm{a}}{\rm{g}}{\rm{e}}\,{\rm{a}}{\rm{t}}\,{\rm{w}}{\rm{e}}{\rm{e}}{\rm{k}}\,({\rm{n}}+1)-{\rm{V}}{\rm{e}}{\rm{s}}{\rm{s}}{\rm{e}}{\rm{l}}{\rm{s}}\,{\rm{d}}{\rm{e}}{\rm{n}}{\rm{s}}{\rm{i}}{\rm{t}}{\rm{y}}\,{\rm{p}}{\rm{e}}{\rm{r}}{\rm{c}}{\rm{e}}{\rm{n}}{\rm{t}}{\rm{a}}{\rm{g}}{\rm{e}}\,{\rm{a}}{\rm{t}}\,{\rm{w}}{\rm{e}}{\rm{e}}{\rm{k}}\,({\rm{n}}),\end{array}$$

where n = 1 to 7 computed for the OCTA imaging performed at Weeks 1 to 8.

To investigate the repeatability and possible error in the vessel growth measurements, two sets of scans were obtained for each time point, 1–2 minutes apart^53^. The vessel density difference computed for the repeated scans were compared with the values of vessel growth density at consecutive follow-ups (1 week apart) for significant difference (Fig. 6). All image processing was performed with MATLAB R2017a (The MathWorks, Inc., Natick, Massachusetts, United States).

### Statistical analysis

The vessel density measurements of all imaging techniques were checked for normality using the Kolmogorov-Smirnov Test and calculated for mean and standard deviation (SD). Mean differences of the measurements were checked for significance using a paired T-test. Agreement of the vessels density measurements between each imaging technique was described using Bland-Altman analysis. The 95% limit of agreement (LoA) and mean difference ± 1.95 SD with 95% CI were calculated using MedCalc Version 17.1. The statistical tests were performed with the Statistical Package for Social Science (SPSS, Chicago, IL, US), version 20; statistical significance was considered *P* < 0.05.

## Electronic supplementary material

Supplementary Graph
