# Supplementary material for: Comparison of Optical Coherence Tomography Angiography to Indocyanine Green Angiography and Slit Lamp Photography for Corneal Vascularization in an Animal Model
Source: Sci Rep. 2018 Jul 31;8:11493. doi: 10.1038/s41598-018-29752-5 (PMC6068177; doi:10.1038/s41598-018-29752-5)

Title: Comparison of Optical Coherence Tomography Angiography to Indocyanine Green Angiography for Corneal Vascularization in an Animal Model

Authors: Tisha P. Stanzel[1], Kavya Devarajan[1] , Nyein C. Lwin[1], Gary H. Yam[1,2], Leopold Schmetterer[1,2,3,4,5], Jodhbir S. Mehta[1,2,3,6], Marcus Ang[1,2,6, *]

Supplementary Graph: Vessels density measurements from indocyanine green angiography (ICGA) compared with slit lamp photography (SLP)

Good agreement of vessel density measurements between ICGA and SLP was observed with mean difference of 0.02 ± 0.07 % (95%CI: - 0.0413 to 0.0053 %), P = 0.11 (10 sets of paired images)

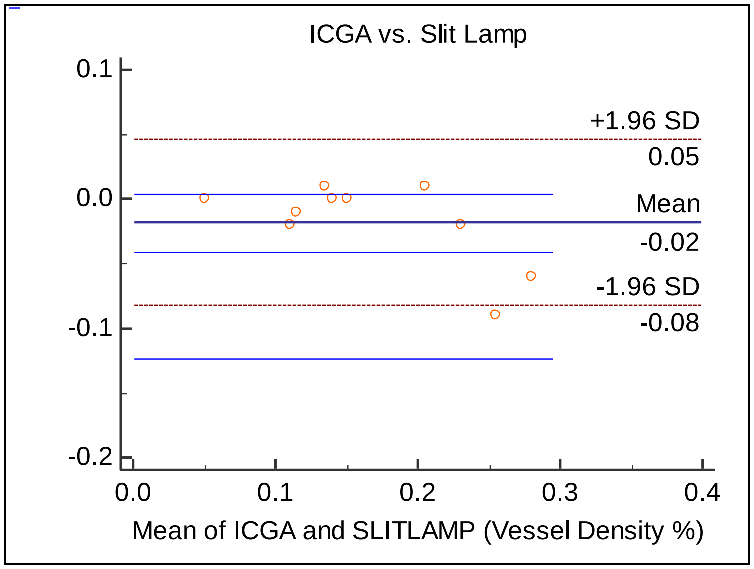

Supplement: Supplementary file 1 — Supplementary Graph [file 41598_2018_29752_MOESM1_ESM.pdf]
